# Visualization of Concrete Slump Flow Using the Kinect Sensor

**DOI:** 10.3390/s18030771

**Published:** 2018-03-03

**Authors:** Jung-Hoon Kim, Minbeom Park

**Affiliations:** 1 Construction Robot and Automation Laboratory, Department of Civil & Environmental Engineering, Yonsei University, Seoul 03722, Korea; minbeom127@gmail.com; 2 Engineering & Construction Group, Samsung C&T Corporation, Seoul 13530, Korea

**Keywords:** visualization of flowable concrete, 3D depth sensor, Kinect, digital image processing, coordinate transform, slump flow test, concrete workability test, structural health monitoring during construction, automated measurement in construction

## Abstract

Workability is regarded as one of the important parameters of high-performance concrete and monitoring it is essential in concrete quality management at construction sites. The conventional workability test methods are basically based on length and time measured by a ruler and a stopwatch and, as such, inevitably involves human error. In this paper, we propose a 4D slump test method based on digital measurement and data processing as a novel concrete workability test. After acquiring the dynamically changing 3D surface of fresh concrete using a 3D depth sensor during the slump flow test, the stream images are processed with the proposed 4D slump processing algorithm and the results are compressed into a single 4D slump image. This image basically represents the dynamically spreading cross-section of fresh concrete along the time axis. From the 4D slump image, it is possible to determine the slump flow diameter, slump flow time, and slump height at any location simultaneously. The proposed 4D slump test will be able to activate research related to concrete flow simulation and concrete rheology by providing spatiotemporal measurement data of concrete flow.

## 1. Introduction

Monitoring the workability of freshly mixed concrete is necessary to ensure that the concrete is properly placed during construction and that adequately hardened strength is achieved after construction [1]. Both are related to structural integrity, safety, and construction productivity. For the safe and easy construction of high-quality concrete structures, it is necessary to use fresh concrete with proper workability. Workability such as flowability, viscosity, yield stress, and resistance to material separation required at construction sites depends on the type of target structure, the spacing of reinforcements, dimensions, cross-sectional shape, construction method, and concrete pumping distance [2]. Monitoring of concrete workability [3,4], as well as strength [5,6,7], is regarded as a key technology in the long-distance transport of concrete required for high-rise buildings and long-span bridge construction. In the field of innovative concrete 3D printing technology in the construction area, workability control will be critical to ensure consistent material properties and structural performance [8,9,10].

In the field of concrete materials, highly flowable self-consolidating concrete (SCC) is attracting attention for its ability to fill congested rebar spaces in a formwork under its own weight without using a vibrator. The characteristics of SCC are filling ability, passing ability, and stability [11]. The use of SCC can increase the construction efficiency because it can improve the quality of a concrete structure, reduce the labor requirement, and increase the construction speed. The use of SCC is expected to increase as the complexity of building design and the number of high-rise buildings increase [4].

**Figure 1:**
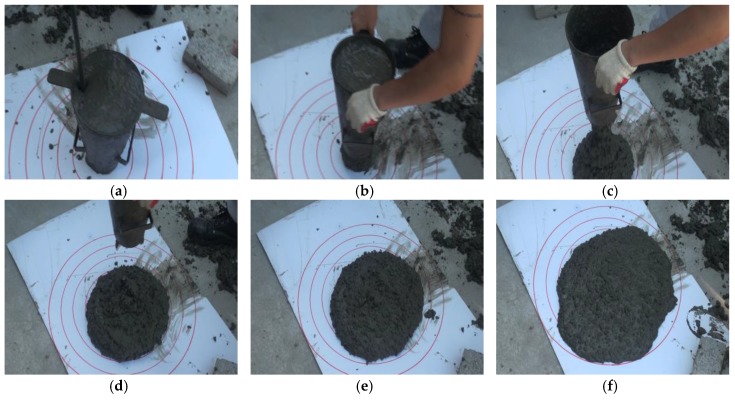
Slump flow test: (**a**) Slump cone filled with fresh concrete; (**b**) Start of slump flow test; (**c**) Flow of concrete from cone; (**d**) Concrete slump after lifting up cone; (**e**) Spreading concrete slump; (**f**) Final concrete slump.

As a standard test method to assess the filling ability of flowable concrete, the slump flow test shown in Figure 1 has been established [12,13,14]. It is the simplest and most widely used test method for SCC [1]. The result of the slump flow test is recorded by measuring the diameter of the circular spread of the concrete after lifting up the slump cone. As non-mandatory information, relative viscosity can be measured during the slump flow test [13]. The T50 value indicating the relative viscosity is the time for the outer edge of the spreading concrete mass to reach a diameter of 500 mm. Most test methods, such as the slump flow test and the slump test [15], are carried out by operators using a ruler or a stopwatch, and hence measurement errors are inherent and the measured value may vary depending on the operator. There is also a possibility that the obtained value can be wrongly recorded or easily manipulated after measurement. If concrete with insufficient workability is used for the construction of structures because of inaccurate measurement results or incorrect data records, there will be future problems in terms of structural safety.

With the development of SCC and high-performance concrete, instead of the conventional test devices, a rheometer has been introduced to measure the rheological parameters of concrete [16]. For example, it has been used for workability monitoring of high-rise buildings [3,4]. Recently, concrete flow based on a computational fluid dynamics (CFD) analysis has been actively studied with a rheometer [17,18]. However, there is a lack of understanding about concrete rheology in the construction field. Furthermore, with the price of a rheometer ranging from $20,000 to $180,000, its use in the field is limited by its costliness. The workability measuring equipment required in the construction field thus should be able to quantitatively evaluate the workability at low cost.

**Figure 2:**
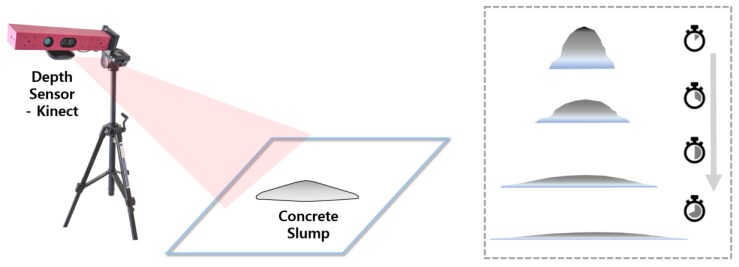
Utilization of the depth sensor during the slump flow test; recording and processing of a dynamically changing surface of fresh concrete.

In this study, a new framework for qualitatively evaluating concrete workability using a depth sensor is proposed. Kinect, a low-cost depth sensor, is utilized to measure the dynamically changing 3D concrete surface during a slump flow test, as shown in Figure 2. The Kinect was originally developed for human motion recognition in the X-box, a game console developed by Microsoft Corporation [19]. Its resolution and its scanning speed are excellent and due to mass production it is inexpensive. Its use is expanding in various fields, such as robotics [20,21], rehabilitation engineering [22], and even in the construction field in monitoring structural health [23,24]. Visualization of spatiotemporal data using sensors is useful for numerical analysis. Just as the infrared thermography is useful for heat transfer analysis of concrete surfaces [25], the 4D slump test using Kinect will activate research on concrete flow simulation.

This paper is organized as follows. Section 2 presents the data processing algorithm, related to the workability test, which can produce the spreading diameter over time and slump flow time from the Kinect data. The data acquired in the depth sensor are represented in the coordinate frame on the ground plane by coordinate transformation, which is well used in computer graphics and robot kinematics. The transformed point cloud data are then used to create the surface of the concrete and the surface is reorganized in a grid form for further data processing. A final 4D slump image is constructed by collecting the extracted cross-sections of the concrete slump at each instant surface image. In Section 3, the experimental setup for 4D slump, including the test material and procedure, is described. In Section 4, the experimental results show that concrete flow visualization is possible during the slump flow test by processing the time-varying surface shape of the concrete.

## 2. Data Processing Algorithm for the 4D Slump Test

The Kinect is used to acquire spatial information of the dynamically changing surface of fresh concrete during the concrete slump flow test. The raw data cannot be directly utilized to analyze useful information on the concrete workability; several stages of data processing are needed to produce slump flow diameter, slump flow time, and slump height. The proposed algorithm for data processing consists of the following procedures: (1) representation of 3D spatial information at the camera frame {C} from the depth image; (2) determination of a ground plane equation; (3) calculation of a transformation matrix between the camera frame {C} and the slump frame {S} where the slump cone is initially located; (4) reconstruction of the 3D surface for concrete slump; and (5) cross-section extraction and construction of a 4D slump image. Each procedure is described in the following sections.

### 2.1. Representation of 3D Spatial Information at Camera Frame {C}

**Figure 3:**
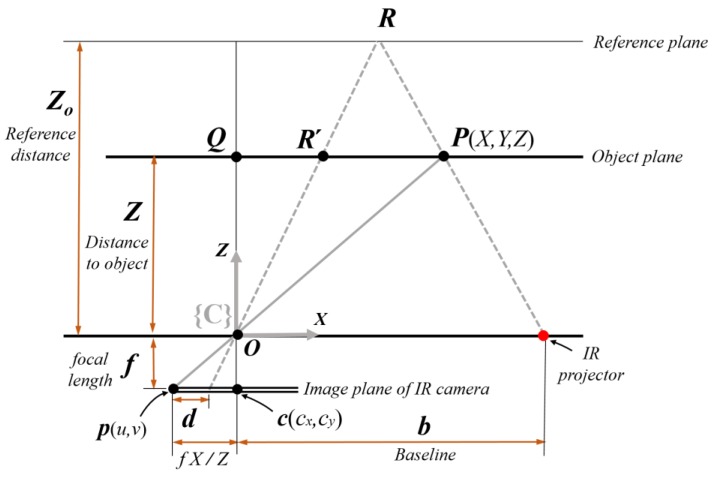
Depth measurement geometry in the pinhole camera model.

While projecting a known speckle pattern of near-infrared light, the Kinect sensor acquires disparity images at the IR camera in real time [26], where the disparity *d* is represented at the pixel location (*u*, *v*). Figure 3 represents the pinhole camera model, which shows how the point in three-dimensional space is projected onto the image plane of the IR camera. The point *P*(*X*,*Y*,*Z*) described in the camera coordinates is projected to the point *p*(*u*,*v*) on the image plane, which is apart from the camera frame by a focal length *f*. Here, the coordinate system of (*X*,*Y*,*Z*) is referenced to the camera frame {C}. Using the proportionality of two similar triangles *OPQ* and *Opc*, the following equations for coordinates *X* and *Y* are obtained:

(1) $$X=\frac{\left(u-c_{x}\right)Z}{f}\;Y=\frac{\left(v-c_{y}\right)Z}{f}$$

where *f* is focal length and *c_x_* and *c_y_* are optical center pixels in the IR camera. The focal length and optical center pixels can be determined by a standard calibration of the camera.

Depth *Z* is expressed as a nonlinear function of disparity *d* [21]. The mathematical model between depth and disparity *d* is derived from the geometric relationship in Figure 3, which is given by Equation (2) [27]:

(2) $$Z=\frac{Z_{0}}{1+\frac{Z_{0}}{fb}d}$$

where *Z*_0_ is the distance to the reference plane, *b* is the baseline between the IR projector and camera. Since the output value by the sensor is the normalized disparity *d*’ in practice, which ranges from 0 to 2^11^ − 1, *d* should be replaced with *md*’ + *n* [27,28] and then Equation (2) becomes as follows:

(3) $$\frac{1}{Z}=\left(Z_{0}^{-1}+\frac{n}{fb}\right)+\left(\frac{m}{fb}\right)d^{\prime }=C_{0}+C_{1}d^{\prime }=\left[\begin{array}{cc} 1 & d\prime \end{array}\right]\left[\begin{array}{c} C_{0}\\ C_{1} \end{array}\right]$$

Equation (3) includes five parameters such as *Z*_0_, *b*, *f*, *m*, and *n* but depth *Z* can be determined by two parameters, *C*_0_ and *C*_1_. These parameters should be estimated by depth calibration. In the calibration experiment, several sets of disparity information and the measured depth are collected. Since the inverse of depth has a linear relationship with the disparity in Equation (3), the calibration parameters *C*_0_ and *C*_1_ can be calculated in terms of pseudo-inverse form by the least square method. By calibrating the parameters, the systematic error due to *Z*_0_, *b*, and *f* can be eliminated and depth accuracy can be improved [28].

To sum up, the final coordinates of *X*, *Y*, *Z* for the given (*u*,*v*,*d’*) are calculated as follows:

(4) $$\begin{array}{ccc} X=\frac{\left(u-c_{x}\right)Z}{f}, & Y=\frac{\left(v-c_{y}\right)Z}{f}, & Z= \end{array}\frac{1}{C_{0}+C_{1}d\prime }$$

Using the above equations with the calibration parameters, it is possible to reconstruct the points in 3D coordinates again from the disparity image.

### 2.2. Determination of the Ground Plane Equation

**Figure 4:**
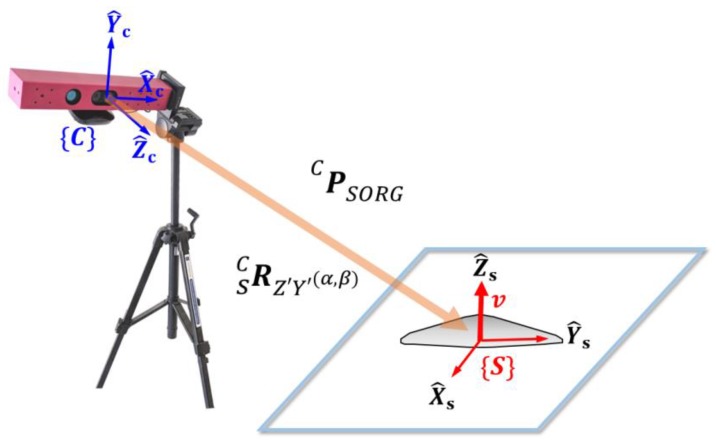
Kinect camera frame {C} and slump frame {S}. *v* is the unit vector on the *Z*-axis of slump frame {S}.

During the slump flow test, the surface of the concrete is detected by a Kinect depth sensor, as shown in Figure 4. There are two coordinate systems, {C} and {S}. {C} is the camera frame where the Kinect IR camera is located and {S} is the slump frame where the concrete slump is located. In order to represent the points P(*X*,*Y*,*Z*) in the camera frame {C}, the expression of *^C^P* is used in this paper. Here, the leading superscript indicates the coordinate system to which the points *P* are referenced. For the data processing of the measured point cloud, it is convenient to use a description of the points in frame {S}, that is, *^S^P*. In order to change the description from frame {C} to {S}, the coordinate transformation can be adopted as a mathematical tool. The theory of coordinate transformation is well established in the field of robot kinematics to express the position and orientation of a robot’s end effector, which is serially connected by several links and joint angles [29]. As the first step, the slump frame {S} should be mathematically represented in the camera frame {C}.

In Figure 4, the slump frame {S} is defined as a frame whose *xy* plane is the ground plane and whose *Z*-axis is the opposite direction of gravity. In order to transform *^C^P* to *^S^P*, the relative rotation matrix RSC and translation vector *^S^P**_SORG_*** are required. In order to calculate the rotation matrix RSC, it is basically necessary to obtain the plane equation of the ground surface where the slump test is performed. 

In this paper, the RANSAC (RANdom SAmple Consensus) algorithm [30] is utilized to obtain the ground plane equation. This algorithm is an iterative method to estimate parameters of a mathematical model from a set of observed data containing outliers. Here, it is used to estimate the parameters (*a*, *b*, *c*, and *d*) of the following equation from a set of selected data on the ground plane around the slump.

(5) aX+bY+cZ+d=0

The unit vector of the *Z*-axis in frame {S} is the orthonormal vector of the ground plane. It is defined as *v* in this paper and is expressed as the parameters of the ground plane equation as follows:

(6) $$v=v_{x}\hat{i}+v_{y}\hat{j}+v_{z}\hat{k}=\frac{a}{\sqrt{a^{2}+b^{2}+c^{2}}}\hat{i}+\frac{b}{\sqrt{a^{2}+b^{2}+c^{2}}}\hat{j}+\frac{c}{\sqrt{a^{2}+b^{2}+c^{2}}}\hat{k}$$

**Figure 5:**
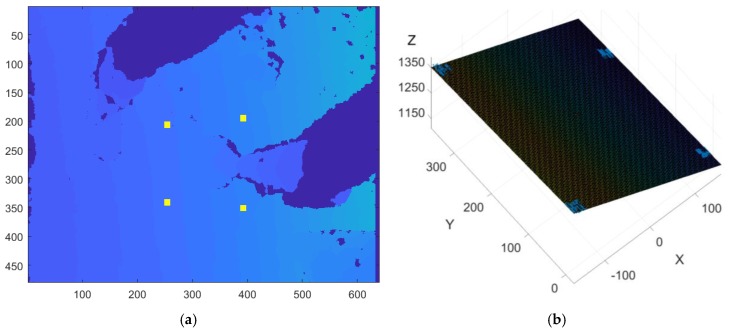
Generation of the ground plane using the RANSAC algorithm: (**a**) Depth image captured by Kinect during a slump test and the selection of four rectangular areas; (**b**) Ground plane generated from the RANSAC algorithm using the selected data.

It is reasonable to use the RANSAC algorithm since it detects outlier data of Kinect and does not use them in fitting the plane equation. That is, unlike the least square method, it is possible to remove the effects of the extreme values resulting from erroneous measurement or environmental conditions such as light intensity or reflectivity. Figure 5a shows the depth image captured in the Kinect during the slump test. If four rectangular areas around the concrete slump are selected, the ground plane is generated by the RANSAC algorithm using the ground points at four rectangular areas, as shown in Figure 5b. The origin point of {S} can be selected as the center of four rectangular areas. The unit vector on the *Z*-axis of the frame {S}, *v* is used to calculate the relative orientation between the camera frame {C} and the slump frame {S} in the next section.

### 2.3. Coordinate Transformation from Camera Frame to Slump Frame

In order to describe the measured points of fresh concrete in slump frame {S}, the transformation matrix between {C} and {S} should be obtained. The relationship between {S} and {C} is characterized by the rotation matrix, RSC, as well as the translational vector, *^C^P_SORG_*. *^C^P_SORG_* is the vector from the camera frame {C} to the slump frame {S}.

**Figure 6:**
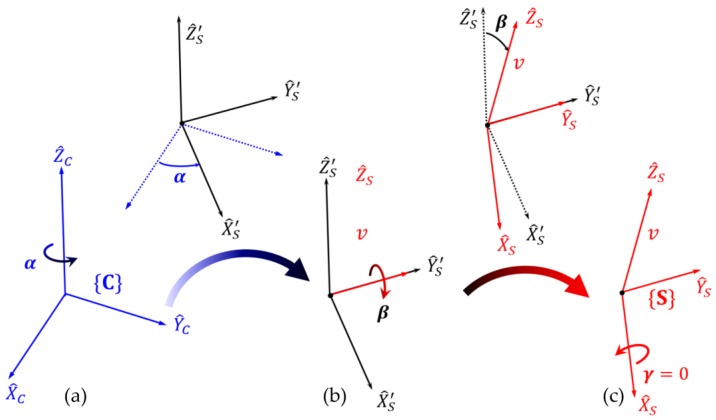
*Z*-*Y*-*X* Euler angles: (**a**) rotation around ${\hat{Z}}_{C}$; (**b**) rotation around ${\hat{Y\prime }}_{S}$; (**c**) rotation around ${\hat{X}}_{S}$.

When an orthonormal vector to the ground plane is represented as $\hat{v}=v_{x}\hat{i}+v_{y}\hat{j}+v_{z}\hat{k}$ in {C}, it is possible to find the rotation matrix of {C} relative to {S} based on the *Z*-*Y*-*X* Euler angles [29]. Any orientation can be achieved by three rotations about the axes of a moving frame in Euler angles. Figure 6 shows the axes of {S} after consecutive Euler angle rotations are applied. When the origin of frame {S} is initially coincident with that of frame {C} in Figure 6, rotation α about ${\hat{Z}}_{c}$ causes ${\hat{X}}_{c}$ to rotate into ${\hat{X\prime }}_{s}$, and ${\hat{Y}}_{c}$ to rotate into ${\hat{Y\prime }}_{s}$. Here, the angle *α* is determined when the vector ${\hat{X\prime }}_{s}$ is located in the plane formed by ${\hat{Z}}_{c}$ and $\hat{v}$. The next rotation is performed about an axis of the intermediate moving frame {S’}. It is possible to find *β* when rotation *β* about ${\hat{Y\prime }}_{s}$ causes $\hat{Z}\prime_{s}$ to rotate into ${\hat{Z}}_{s}$, that is, $\hat{v}$ (orthonormal vector to the ground plane). As a result of two consecutive rotations about the axes of the moving frame, the final orientation of {S} is given relative to {C} as:

(7) RSC=RSCZ′Y′X′(α,β,0)=RZ(α)RY(β)RX(0)= [cα−sα0sαcα0001][cβ0sβ010−sβ0cβ][100010001]=[cαcβ−sαcαsβsαsβcαsαsβ−sβ0cβ]

**Figure 7:**
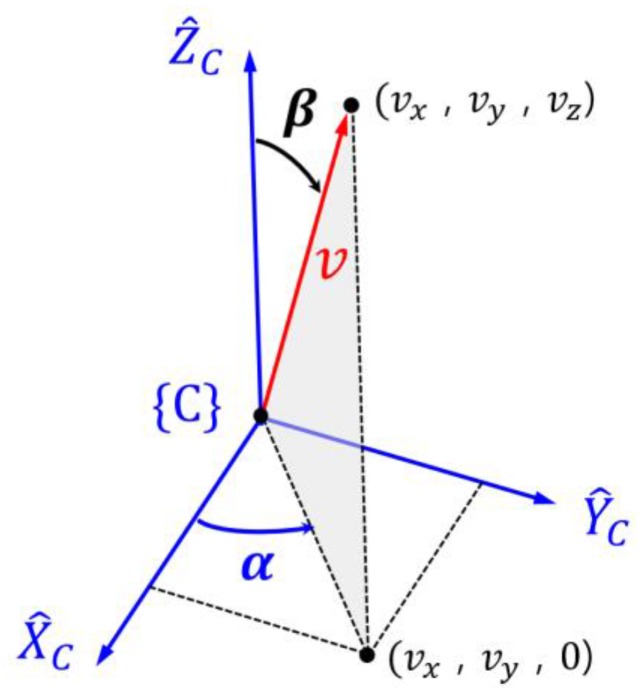
An orthonormal vector *v* perpendicular to the ground plane described in frame {C}.

The angles *α* and *β* can be easily expressed in terms of the components of the orthonormal vector *v* in frame {C}, as illustrated in Figure 7. α represents the angle between $v_{x}\hat{i}+v_{y}\hat{j}$ and the *X*-axis, and *β* refers to the angle between $v_{x}\hat{i}+v_{y}\hat{j}+v_{z}\hat{k}$ and the *Z*-axis. They can be expressed as follows:

(8) $$\alpha =tan^{-1}\left(v_{y},v_{x}\right)\;\beta =acos\left(v_{z}\right)$$

The homogeneous transformation matrix TSC that maps *^S^P* to *^C^P* is represented by the orientation and position information as follows [29]:

(9) TSC= [RSC000 PCSORG1]

The columns of RSC are unit vectors defining the directions of the principal axes of {S} and *^C^P_SORG_* represents the position vector of the origin of {S} in frame {C}. The description of points with respect to the slump frame {S} is calculated by the inverse of TSC as follows:

(10) PS=TCSPCwhere TCS= [(RSC)T000 −(RSC)TPCSORG1]

**Figure 8:**
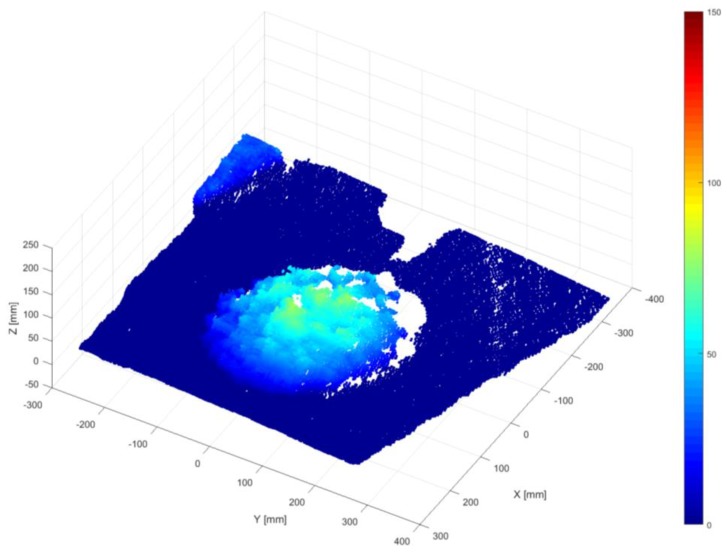
Concrete slump described in frame {S}.

Figure 8 shows the result of the transformation of points in the camera frame to the slump frame using Equation (10).

### 2.4. Reconstruction of 3D Surface for Concrete Slump

**Figure 9:**
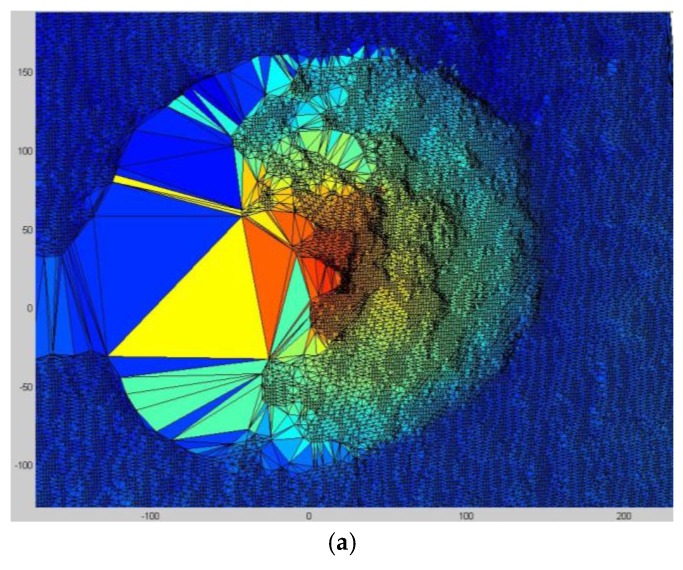

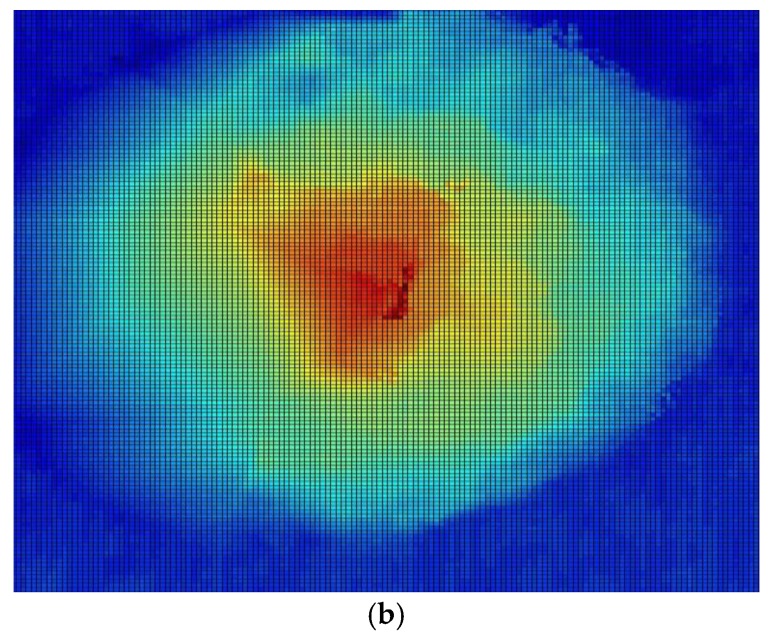
Reconstruction of the 3D surface: (**a**) Surface of the concrete slump generated by the triangulated irregular network (TIN); (**b**) Surface of the concrete slump in grid coordinate (Result of triangulation-based linear interpolation for TIN).

Triangulated irregular network (TIN) is the digital data structure generally used in geographic information systems (GIS) to represent the topographical surface. TIN is composed of contiguous, non-overlapping triangles. It can be used to convert the measured scattered data points into a model of the 3D surface. The surface of slump flow generated by TIN is shown in Figure 9a. Large triangles on the left part of Figure 9a are the shadow areas where shooting is restricted by the angle of the Kinect sensor. The vertexes of TIN are located at irregularly spaced points (*x_i_*, *y_i_*) in the plane, that is, TIN itself does not have equal intervals in the *X-* and *Y*-axes. For ease of data analysis, such as cross-section extraction, it is desirable to construct a network composed of regular grids, as shown in Figure 9b. Triangulation-based linear interpolation is applied to generate data at the grid points [31]. Each triangle plane is defined by three points in the equation *z* = *ax* + *by* + *c*, and any point located at a specific grid (*x*,*y*) within this triangle can be linearly interpolated. In this grid coordinate, it is easy to extract the slump height at a specific location of the ground plane.

### 2.5. Cross-Section Extraction and Construction of a 4D Slump Image

**Figure 10:**
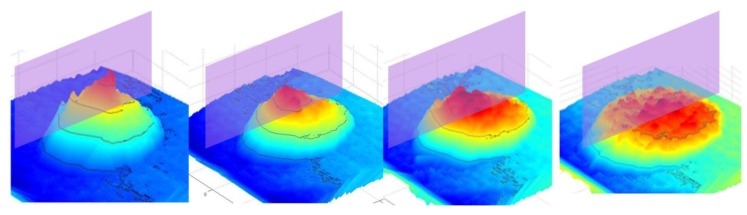
Spreading shape of concrete slump flow with time.

Through the above-mentioned data processing algorithm, the shape information obtained from the depth sensor camera can be expressed in a 3D surface where the height data are available at any grid position of the slump frame {S}. The dynamically changing shapes of the concrete slump over time are the four-dimensional spatiotemporal data, as shown in Figure 10. Here, every image of the dynamically changing slump flow shape is the result of applying the series of data processing procedures described above.

By selectively extracting the information from each slump in Figure 10, it is possible to reconstruct the data and visualize the workability effectively. For example, the cross-sectional curve (2D) of a slump can be collected over time (1D) and reconstructed into three-dimensional data, which again can be compressed into a 2D image, as seen in the top view. In this paper, the compressed 2D image is called a 4D slump image and it will be shown in Section 4 with an explanation of the experiment in Section 3.

## 3. Experimental Setup for the 4D Slump Test

### 3.1. Devices for the 4D Slump Test

As a low-cost 3D depth sensor, Microsoft Kinect was utilized to acquire data during the slump test. Kinect consists of an IR laser pattern projector, an IR camera, and an RGB camera. When the IR projector projects a laser speckle pattern onto an object, an image of the pattern on the object is captured by the IR camera and then processed to reconstruct a 3D map of the object [26]. The Kinect provides a depth video stream with a resolution of 640 × 480 pixels at a maximum of 30 frames per second. At a lower frame rate, it can deliver 1280 × 1024 pixels. The default sensing range is 0.8 m to 6 m but it is configured to near mode, which provides a range of 0.4 m to 3 m for a better quality of resolution [32]. Depth resolution refers to the minimum detectable difference in a certain continuous distance range [33]. Based on research by Smisek et al., the resolution is 0.65 mm at 0.5 m and it changes with the distance in a quadratic manner [19]. The depth resolution is less than 2 mm at 1 m and 25 mm at a 3-m distance [27]. According to the investigation of Khoshelham et al. on the Kinect, the random error of the depth measurement also increases in a quadratic manner as the distance of the object increases [27]. The random error is less than 0.6 cm at a distance of 1 m but it reaches 4 cm at the maximum range of 5 m. During the experiment, the distance was kept to less than 1 m where Kinect had a resolution of 2 mm and a random error of 6 mm. It is not recommended to increase the distance greater than 1 m because the quality of the depth data is degraded by random error and resolution.

**Figure 11:**
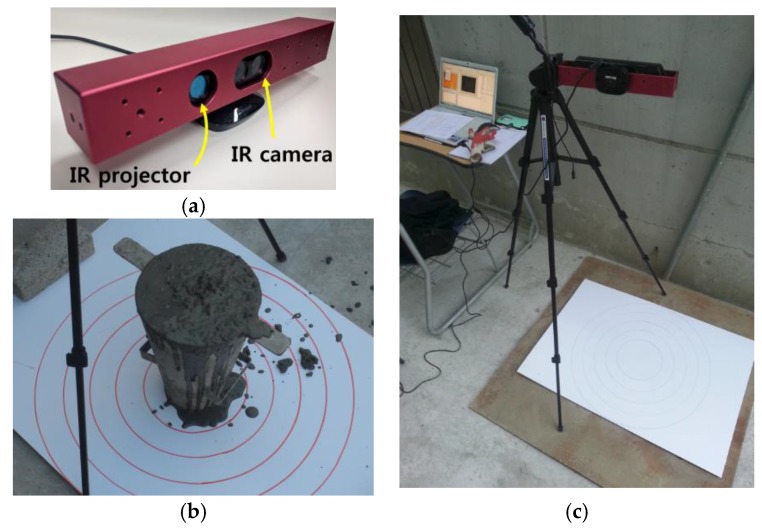
Experimental setup: (**a**) Kinect with camera mounting frame; (**b**) Slump cone in an inverted position; (**c**) Kinect and laptop computer running the 4D slump test program.

The Kinect was assembled with an in-house mounting frame, as shown in Figure 11a. The incorporation of several bolt holes on the mounting frame enabled a better shooting angle for measurement by providing additional freedom to connect it to the camera tripod. The coordinate transform algorithm allowed shooting from any angle with Kinect. Kinect drivers such as OpenNI and NiTE were installed on a Windows 7 laptop computer and an in-house MATLAB code was run for measurement and analysis. 

### 3.2. Test Material and Procedure

As a flowable concrete, an SSC mixture with a water-to-binder ratio of 39% was used in the experiment. The proportions of concrete mix are shown in Table 1. The diameter of the silica sand used in the mix ranged from 0.1 mm to 0.6 mm. The diameter of the coarse aggregate was less than 13 mm.

The slump flow test was performed based on ASTM C1611 [13]. The cone was filled with fresh concrete to the struck volume in the inverted position on a flat, level, non-absorbent surface, as shown in Figure 11b. When the cone was lifted, the concrete flowed out. The slump flow test was recorded in 24 frames per second by a laptop connected to the Kinect sensor while the raw depth data were displayed on the monitor. In ASTM C1611, slump flow and T50 are measured by operators. Slump flow is the average of two perpendicular diameters across the spread of concrete and T50 is the time for the fresh concrete to reach a diameter of 500 mm after lifting the slump cone. In the conventional method, it is hard to start and stop a clock at exactly the correct times while conducting the slump flow test. The slump flow time (T50) in the inverted slump cone orientation generally gives a higher value than in the normal orientation [34,35].

## 4. Experimental Results

**Figure 12:**
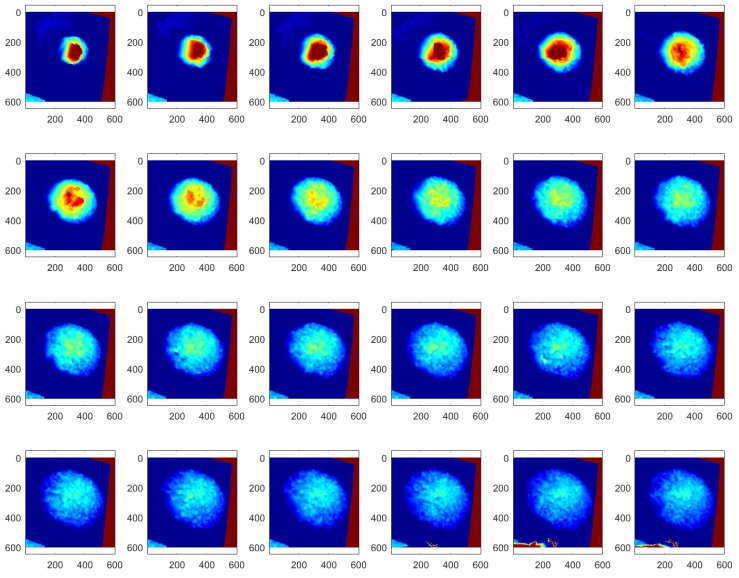
Reconstructed images of the concrete surface over time during the slump flow test (top view).

**Figure 13:**
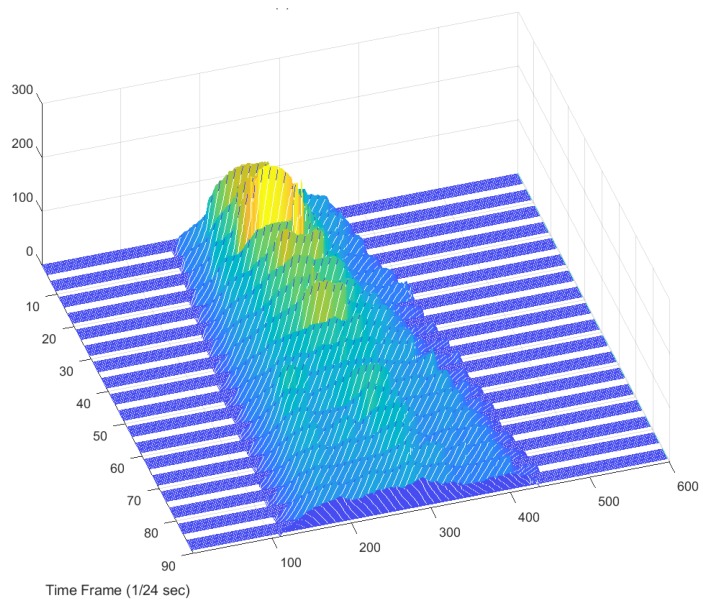
Visualization of spreading concrete cross-section during the slump flow test (3D surface representation).

**Figure 14:**
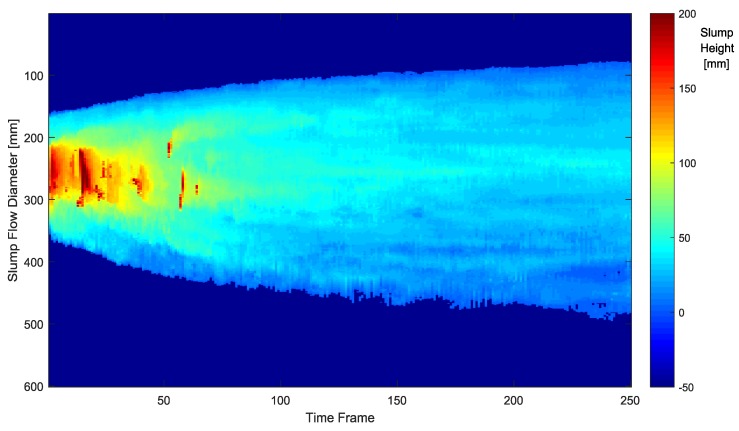
4D slump image (2D color image representation).

The depth images recorded at 24 Hz sampling frequency during the slump flow test were processed by the 4D slump processing algorithm described in Section 2.1, Section 2.2, Section 2.3 and Section 2.4. As a result, reconstructed images of the concrete surface in regularly spaced coordinates of the slump frame at every time frame were obtained, as shown in Figure 12. It was then possible to visualize the spreading cross-section of fresh concrete during the slump flow test, as shown in Figure 13. This 3D surface is made of the extracted 2D cross-sections in a given 3D concrete surface at a specific time frame of Figure 12. By representing the height *z* at a specific location (*x*, *y*) of Figure 13 as color information, the 4D slump image is obtained, as presented in Figure 14. This is a compressed image of the slump flow test showing the dynamically spreading diameter of concrete slump flow. It contains the generalized slump flow time T*i*, the slump diameter at any time, and the height of slump at any time and location. In Figure 14, the local area in red at the initial time frame is the result of the concrete drop from the slump cone.

**Figure 15:**
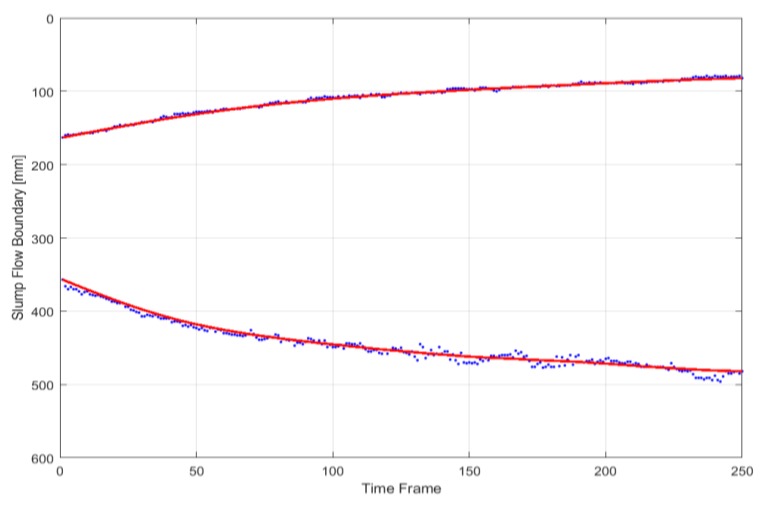
Detected boundaries of slump flow on the 4D slump image.

**Figure 16:**
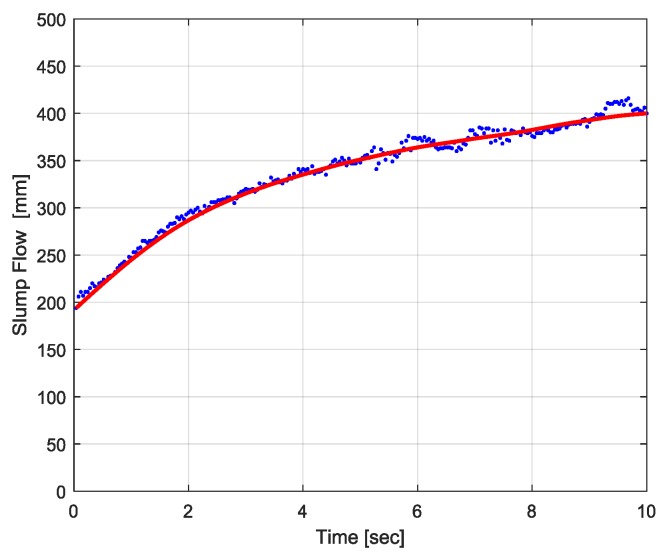
Slump flow diameter over time.

In the 4D slump image, it is possible to detect the boundary of the slump flow, as presented in Figure 15. By utilizing the characteristics of the 4D slump image where the slump flow diameter increases from the left to right side, the boundaries of the slump flow were searched along the vertical axis from the upper and lower border lines to the middle. For an edge detection algorithm, the simple threshold method or Sobel operator could be applied. In Figure 15, the red line represents the moving average of the detected edge points plotted with blue color. After detecting the upper and lower boundaries of the slump flow in the 4D slump image, the slump flow diameter over time was obtained, as shown in Figure 16, by calculating the relative distance between the two boundaries in Figure 15. This graph provides useful information for comparing the experimental behavior of concrete flow with simulation results through a CFD analysis.

## 5. Conclusions

In this paper, a novel workability test method using a 3D depth sensor was proposed and the dynamically spreading diameter of concrete slump flow was successfully visualized through a 4D slump processing algorithm. In order to represent the surface of fresh concrete in the slump frame, we transformed the depth data in the camera frame {C} acquired by Kinect into the data in slump frame {S} with the calculation of the relative orientation and position between the {C} and {S} frames. The scattered data in the slump frame were then interpolated in the regularly spaced grid and the cross-sections of the slump flow at each time frame were collected. Through the experiment, it was confirmed that the information on the spreading slump flow diameter, slump height, and slump flow time could be obtained by a single 4D slump image using the proposed 4D slump processing algorithm. In the conventional concrete workability test device, it is impossible to obtain these kinds of images and graphs.

The proposed 4D slump test method offers several advantages. First, instead of human measurements using a ruler and a stopwatch, which can introduce human error, it can digitize the workability data and quantitatively evaluate the workability at low cost. Second, it affords non-contact, portable digital measurement and provides additional information besides the basic information while performing the existing workability test. Third, measurement of concrete flow will be helpful in the quality monitoring of concrete by providing a digital record that cannot be easily modified. In addition, it will accelerate the development of flowable concrete based on numerical simulations by providing digital data for concrete flow experiments. Beside systematic error, random error, and resolution discussed in this paper, the depth measurement quality is influenced by the light condition and target reflectivity. At a high light intensity, the IR camera cannot detect laser speckles on the object since the relative contrast of the speckles is decreased. That is to say, the signal-to-noise ratio of the reflected pattern is low under sunlight. If the water content of fresh concrete is high or the water in the fresh concrete tends to rise to the surface, speckle patterns projected by an IR projector are not reflected back from the concrete surface. Consequently, the Kinect will create gaps and outliers [27,36]. In terms of its usability, it can be used for a slump test, the most popular workability test, since this test only requires measurement of the static height of the concrete slump.

## Figures and Tables

**Table 1:** Mixture mass proportions of test material used in the experiment.

W/B	Water	C	FA	S	G	SP
39%	9.37%	16.77%	7.18%	35.01%	31.52%	0.345%

C: Ordinary Portland cement, ρ = 3.16, s = 3214 cm^2^/g; FA: Flay ash, ρ = 2.19, s = 3960 cm^2^/g; S: Silica Sand, ρ = 2.65, diameter from 0.1 mm to 0.6 mm; G: Gravel, diameter less than 13 mm; SP: Polycarbonate Superplasticizer.

## References

[1] Koehler E.P., Fowler D.W. (2003). Summary of Concrete Workability Test Methods; Research Report ICAR-105-1.

[2] Popovics S. (1982). Fundamentals of Portland Cement Concrete: Fresh Concrete v. 1: Quantitative Approach.

[3] Kwon S.H., Jang K.P., Kim J.H., Shah S.P. (2016). State of the Art on Prediction of Concrete Pumping. Int. J. Concr. Struct. Mater..

[4] Nehdi M.L. (2013). Only Tall Things Cast Shadows: Opportunities, Challenges and Research Needs of Self-Consolidating Concrete in Super-Tall Buildings. Constr. Build. Mater..

[5] Shin S.W., Yun C.B., Popovics J.S., Kim J.H. (2007). Improved Rayleigh Wave Velocity Measurement for Nondestructive Early-Age Concrete Monitoring. Res. Nondestruct. Eval..

[6] Başyiğit C., Çomak B., Kılınçarslan Ş., Serkan Üncü İ. (2012). Assessment of Concrete Compressive Strength by Image Processing Technique. Constr. Build. Mater..

[7] Rizzo P., Ni X., Nassiri S., Vandenbossche J. (2014). A Solitary Wave-Based Sensor to Monitor the Setting of Fresh Concrete. Sensors.

[8] Lilliman M. (2017). Control of Mortar Rheology for 3D Concrete Printing. Ph.D. Thesis.

[9] Perrot A., Rangeard D., Pierre A. (2016). Structural Built-Up of Cement-Based Materials Used for 3D-Printing Extrusion Techniques. Mater. Struct..

[10] Jeon K.-H., Park M.-B., Kang M.-K., Kim J.-H. Development of an Automated Freeform Construction System and its Construction Materials. Proceedings of the 30th International Symposium on Automation and Robotics in Construction.

[11] Khayat K.H. (1999). Workability, Testing, and Performance of Self-Consolidating Concrete. ACI Mater. J..

[12] Bartos P.J.M., Sonebi M., Tamimi A.K. (2002). Workability and Rheology of Fresh Concrete: Compendium of Tests—Report of RILEM TC 145-WSM.

[13] (2014). ASTM C1611/C1611M-14. Standard Test Method for Slump Flow of Self-Consolidating Concrete.

[14] Kurokawa Y., Tanigawa Y., Mori H., Komura R. (1994). A Study on the Slump Test and Slump-Flow Test of Fresh Concrete. Trans. Jpn. Concr. Inst..

[15] (2015). ASTM C143/C143M-15a. Standard Test Method for Slump of Hydraulic-Cement Concrete.

[16] Koehler E.P., Fowler D.W., Ferraris C.F., Amziane S. (2006). A New, Portable Rheometer for Fresh Self-Consolidating Concrete. ACI Specif. Publ..

[17] Kim J.H., Jang H.R., Yim H.J. (2015). Sensitivity and Accuracy for Rheological Simulation of Cement-Based Materials. Comput. Concr..

[18] Gao J., Fourie A. (2015). Spread is Better: An Investigation of the Mini-Slump Test. Miner. Eng..

[19] Smisek J., Jancosek M., Pajdla T., Fossati A., Gall J., Grabner H., Ren X., Konolige K. (2013). 3D with Kinect. Consumer Depth Cameras for Computer Vision: Research Topics and Applications.

[20] El-laithy R.A., Huang J., Yeh M. Study on the Use of Microsoft Kinect for Robotics Applications. Proceedings of the 2012 IEEE/ION Position, Location and Navigation Symposium.

[21] Park J.-H., Shin Y.-D., Bae J.-H., Baeg M.-H. (2012). Spatial Uncertainty Model for Visual Features Using a Kinect™ Sensor. Sensors.

[22] Tannous H., Istrate D., Benlarbi-Delai A., Sarrazin J., Gamet D., Ho Ba Tho M., Dao T. (2016). A New Multi-Sensor Fusion Scheme to Improve the Accuracy of Knee Flexion Kinematics for Functional Rehabilitation Movements. Sensors.

[23] Qi X., Lichti D., El-Badry M., Chow J., Ang K. (2014). Vertical Dynamic Deflection Measurement in Concrete Beams with the Microsoft Kinect. Sensors.

[24] Mohamed A., Yulu Luke C., Mohammad R.J., Sami F.M., Wei-Men S., Uvais A.Q. (2017). 3D Dynamic Displacement-Field Measurement for Structural Health Monitoring Using Inexpensive RGB-D Based Sensor. Smart Mater. Struct..

[25] Khan F., Bolhassani M., Kontsos A., Hamid A., Bartoli I. (2015). Modeling and experimental implementation of infrared thermography on concrete masonry structures. Infrared Phys. Technol..

[26] Freedman B., Shpunt A., Machline M., Arieli Y. (2013). Depth Mapping Using Projected Patterns. U.S. Patent.

[27] Khoshelham K., Elberink S.O. (2012). Accuracy and Resolution of Kinect Depth Data for Indoor Mapping Applications. Sensors.

[28] Darwish W., Tang S., Li W., Chen W. (2017). A New Calibration Method for Commercial RGB-D Sensors. Sensors.

[29] Craig J.J. (2004). Introduction to Robotics: Mechanics and Control.

[30] Fischler M.A., Bolles A.R.C. (1981). Random Sample Consensus: A Paradigm for Model Fitting with Applications to Image Analysis and Automated Cartography. Commun. ACM.

[31] Amidror I. (2002). Scattered Data Interpolation Methods for Electronic Imaging Systems: A Survey. J. Electron. Imaging.

[32] Kinect Sensor. https://msdn.microsoft.com/en-us/library/hh438998.aspx.

[33] Yang L., Zhang L., Dong H., Alelaiwi A., Saddik A.E. (2015). Evaluating and Improving the Depth Accuracy of Kinect for Windows v2. IEEE Sens. J..

[34] Fares G. (2015). Effect of Slump Cone Orientation on the Slump Flow Time (T50) and Stability of Sustainable Self-Compacting Concrete Containing Limestone Filler. Constr. Build. Mater..

[35] Ramsburg P. (2003). The SCC Test: Inverted or Upright?. Concr. Prod..

[36] Azzari G., Goulden L.M., Rusu B.R. (2013). Rapid Characterization of Vegetation Structure with a Microsoft Kinect Sensor. Sensors.

